# Application of Strategic Transport Model and Google Maps to Develop Better Clot Retrieval Stroke Service

**DOI:** 10.3389/fneur.2019.00692

**Published:** 2019-06-28

**Authors:** Atousa Tajaddini, Thanh G. Phan, Richard Beare, Henry Ma, Velandai Srikanth, Graham Currie, Hai L. Vu

**Affiliations:** ^1^Department of Civil Engineering, Institute of Transport Studies, Monash University, Melbourne, VIC, Australia; ^2^Stroke Unit, Monash Health, Melbourne, VIC, Australia; ^3^Stroke and Aging Research Group, Medicine, School of Clinical Sciences, Monash University, Melbourne, VIC, Australia; ^4^Department of Medicine, Frankston Hospital, Peninsula Health, Melbourne, VIC, Australia; ^5^Central Clinical School, Monash University, Melbourne, VIC, Australia; ^6^Developmental Imaging, Murdoch Children's Research Institute, Melbourne, VIC, Australia

**Keywords:** stroke, transport, optimization, Google Maps, endovascular clot retrieval

## Abstract

**Background and purpose:** Two hubs are designated to provide endovascular clot retrieval (ECR) for the State of Victoria, Australia. In an earlier study, Google Maps application programming interface (API) was used to perform modeling on the combination of hospitals optimizing for catchment in terms of current traveling time and road conditions. It is not known if these findings would remain the same if the modeling was performed with a large-scale transport demand model such as Victorian Integrated Transport Model (VITM). This model is developed by the Victorian State Government Transport has the capability to forecast travel demand into the future including future road conditions which is not possible with a Google Maps based applications. The aim of this study is to compare the travel time to potential ECR hubs using both VITM and the Google Maps API and model stability in the next 5 and 10 years.

**Methods:** The VITM was used to generate travel time from randomly generated addresses to four existing ECR capable hubs in Melbourne city, Australia (i.e., Royal Melbourne Hospital/RMH, Monash Medical Center/MMC, Alfred Hospital/ALF, and Austin Hospital/AUS) and the optimal service boundaries given a delivering time threshold are then determined.

**Results:** The strategic transport model and Google map methods were similar with the *R*^2^ of 0.86 (peak and off peak) and the Nash-Sutcliffe model of efficiency being 0.83 (peak) and 0.76 (off-peak travel). Futures modeling using VITM found that this proportion decreases to 82% after 5 years and 80% after 10 years. The combination of RMH and ALF provides coverage for 74% of cases, 68% by 5 years, and 66% by 10 years. The combination of RMH and AUS provides coverage for 70% of cases in the base case, 65% at 5 years, and 63% by 10 years.

**Discussion:** The results from strategic transport model are similar to those from Google Maps. In this paper we illustrate how this method can be applied in designing and forecast stroke service model in different cities in Australia and around the world.

## Introduction

The successes of endovascular clot retrieval (ECR) trials in 2015 (1–6) have generated optimism in the treatment of stroke and also debate on translating of these trials into clinical practice for both rural and metropolitan patients (7). These issues include whether patients should be transported to transfer directly to “mothership” or treat at the local hospital first, so called “drip and ship” (8, 9). Initial management at the local hospital has been associated with delayed onset to revascularization (10) and poorer outcome (11). Such idea on treatment exist previously in the development of primary stroke center (PSC) and comprehensive stroke center (CSC) (12, 13). Hospitals certified as CSC have faster time to reperfusion than PSC (14); these ideas now have taken center stage given the better outcome for ECR in centers with high volume output of cases. However, transfer of all cases or screened positive LVO cases can impact on capacity of the receiving hospital. The capacity of the “mothership” hospital to handle the diversion of patients has not been evaluated. In 2017, it has been estimated that 10–16% of patients would be eligible for ECR. This number will change with the publications of two ECR trials which extend the time window to 16–24 h (15, 16).

The State of Victoria had deemed in 2016 that two ECR hubs would be required for this purpose and performed a rigorous process to select the ECR hubs (17). This idea is similar to the concept of CSC but with a difference that the CSC provide care for the catchment and also outlying rural areas (12). Royal Melbourne Hospital (RMH) was selected as the first site with Monash Medical Center (MMC) added in the year 2018. An initial study showed that the combination of RMH and MMC would be optimal in terms of the ability of patients to travel to these hospitals within the idealized time of 30 min (18). This study was performed using an interface to the Google Maps API to query traveling time at different times of the day. A potential drawback of that study is that it cannot assess stability of the transport model in the future given population growth, increasing number of cars on the road and building of new road links and public transport routes. In this study, a trip-based travel demand model developed for the whole state of Victoria was used to obtain the travel time from a random generated address to each of the nominated ECR-capable hospitals in Melbourne. This method of analysis is standard within the transport industry but is not so well known in the medical literature, Historically, models of these systems have been developed to model the movement patterns of passengers and vehicles in cities. These models are used by transport planners and decision makers to understand the travel behavior of travelers over time (19). The aim of this study is to employ a strategic transport model to evaluate the findings from the Google Maps API and assess if the catchment for the two hospitals remain stable into the future. Consistent with the idea developed in the call for paper in this special issue of Frontiers in Neurology, we will spend the next section discussing how investigators can apply similar methods at their local sites.

## Methodology

### Setting

Melbourne is the second largest city in Australia and is the capital city of the state of Victoria in Australia with a population of approximately 4 million. The addresses were generated from the postcodes for metropolitan Melbourne are in the range 3,000–3,207. This aspect had been described in our earlier paper in 2017 (18).

### ECR Capable Hospitals

There are 4 ECR capable hospitals in Victoria: Royal Melbourne Hospital (RMH), Monash Medical Center (MMC), Austin Hospital (AUS) and Alfred Hospital (ALF). At the time of the writing of the Statewide Protocol for ECR in 2017, it was planned to operate with 2 ECR hubs (17). RMH is located near to the center of Melbourne, MMC to the South-East, AUS to the North and ALF is located between RMH and MMC.

### Transport Modeling

In this paper, an idealized time of 30 min is used based on the modeling in the redesign of stroke service in London (20). In this section, we explain the VITM model as a transport demand model as well as its functionality to generate the service boundaries of nominated ECR-hub in different combinations based on travel time. The Victorian Integrated Transport Model (VITM) is a large-scale trip-based model known as “four-step” process which has been used by the Victorian Department of Transport (DoT) and VicRoads to evaluate the impacts of alternative transportation and land use investments as well as presenting any changes in travel demand in response to different input assumptions (21). This process has four basic phases as its name implies: trip generation, trip distribution, mode choice and, trip assignment (22). This study consists of two main stages. The first stage is to validate the VITM model by comparing the VITM base case 2016 results with travel time data produced by the Google Maps API from the previous study. To this end, different statistical tests such as *R*^2^, RMSE, and NSE will be applied. Once the validity of the VITM model is confirmed, VITM will then be utilized to predict travel time in projection years of 2021 and 2026.

## VITM Model

Trip generation predicts the number of trips produced in a certain area of the network by trip purpose and destined for a particular traffic analysis zone. Trip distribution connects trip production and attraction. Mode choice defines if trip is done with personal vehicle or public transport while trip assignment estimates the specific route for each trip. The original VITM was developed based on the travel data collected during 1990 but recalibrated using the Victorian Integrated Survey of Travel and Activity (VISTA) data (23). VISTA is a household survey diary data of randomly selected households (23). In this data, all information about how individuals travel including a simple walk with their dog to the way they travel between states are gathered. The main goal of this survey is to understand the complex travel behavior of individuals. The model then incorporates the complex interactions within the transport system (e.g., car driving, public transport or other mobility modes) and that with economics, demographic and future land use change. The VISTA data was used in recalibration process to update trip generation, distribution and mode choice modules.

The state-wide version of VITM covers the entire state of Victoria. This model is based on a zone structure which collectively represent the geography of the modeled area. This model consists of 6,973 transport zones (12). The standard outputs from VITM are available at 5-yearly intervals from the latest VISTA data of 2016 year to a 30-year horizon (2046). This model provides travel demand estimates based on trip origin to destination, selected mode of car or public transport for all travel purposes. The car “skim” matrices produced by VITM represent travel time in minutes by time of day period as well as travel distance in form of kilometer by time of day.

### Comparison of Different Models

**Figure 1:**
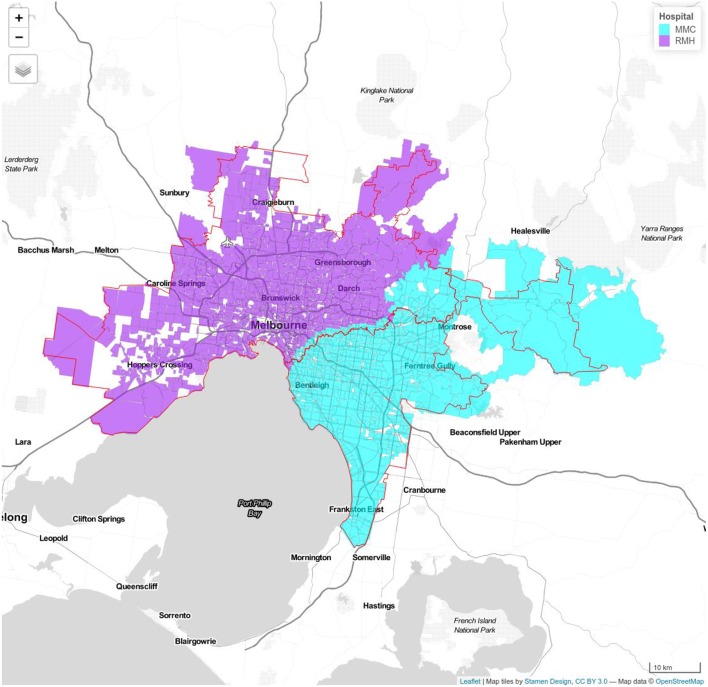
Model 1a Royal Melbourne Hospital (RMH) and Monash Medical Center (MMC). Royal Melbourne Hospital's catchment has purple color and Monash Medical Centre's catchment is displayed with blue color. Red line shows the boundary determined using Google APIs.

**Figure 2:**
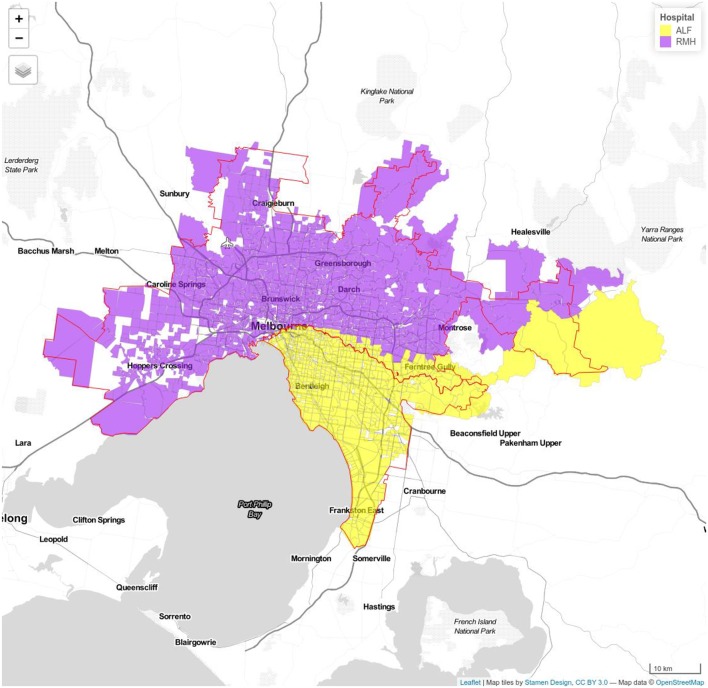
Model 1b Royal Melbourne (RMH) and Alfred Hospitals (ALF). Royal Melbourne Hospital's catchment has purple color and Alfred Hospital catchment is displayed with yellow color.

**Figure 3:**
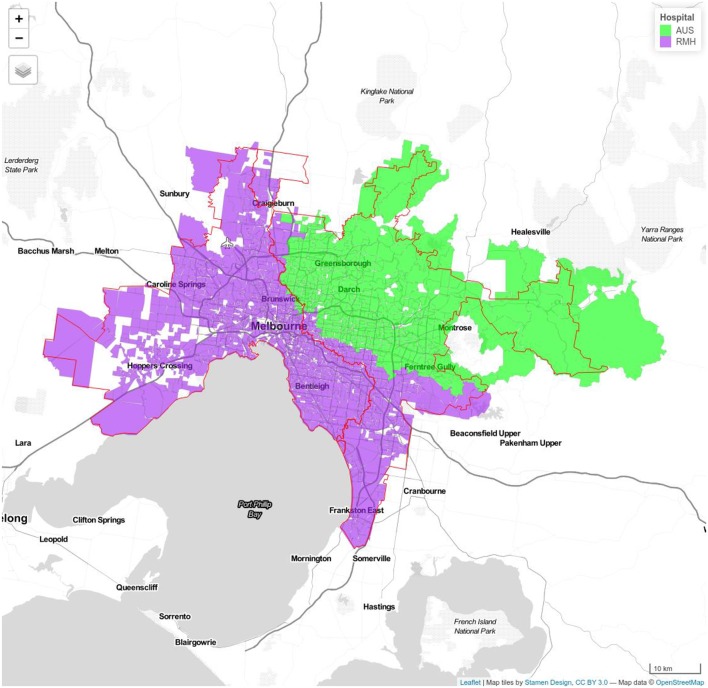
Model 1c Royal Melbourne (RMH) and Austin Hospitals (AUS). Royal Melbourne Hospital's catchment has purple color and Austin Hospital's catchment is displayed with green color.

Traffic zones containing the random addresses used in our previous study were identified, and travel time between each traffic zone and each hospital calculated using the VITM model. The catchment area for each hospital was determined by assigning each traffic zone to the closest hospital according to travel time. To estimate the catchment area of each hospital in 2-hub combinations, the number of zones which have travel time to that hospital less than the paired one were collected. The traveling time to 2-ECR combinations extracted from VITM in comparison to the Google Maps API data as well as the proportion of patients arriving to nominated hospital in each model during period are illustrated in Table 1. Figures 1–3 show the catchment area of RMH as reference hospital in different combination with other hospitals.

**Table 1:** Proportion of patients arriving within 30 min in 2-hub models over base and future years.

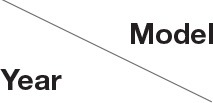	Model 1-a (RMH-MMC) (%)	Model 1-b (RMH-ALF) (%)	Model 1-c (RMH-AUS) (%)	
2016	82	65	63	Peak
2021	79	61	60	
2026	77	59	59	
** 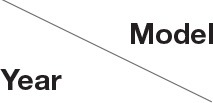 **	**Model 1-a**	**Model 1-b**	**Model 1-c**	
2016	86	74	70	Off peak
2021	82	68	65	
2026	80	66	63	

The findings from Google Map were compared to that by VITM model using the *R*^2^, and Nash–Sutcliffe model efficiency coefficient. The base case refers to the travel times extracted using Google APIs for Wednesday, 8th of June 2016 (24). The R-squared (*R*^2^), and Nash-Sutcliffe model efficiency (NSE) are normally employed in model evaluation studies. *R*^2^ values are within the range of 0 and 1 where values close to 0 show a poor fit and values close to 1 represent a perfect fit. The Nash-Sutcliffe model efficiency coefficient ranges from –∞ to 1. An efficiency of 1 (*NSE* = 1) corresponds to a perfect match of the model (13, 14).

### Stability of the Model in Future Year 2021 and 2026

Input variables to VITM for future years (2021 and 2016) consist of changes in land use data and generalized highway cost calculation including demographic, income growth, vehicle operating cost, parking cost, and parking boundaries. Following we will present results for the permutation of 2-hub in future years. Average time to each hospital in each combination as well as changes in proportion of patients arriving the hubs in critical 30 min during 10 years from 2016 to 2026 are presented inrea (Table 1).

## Results

For travel time forecasts, the strategic transport model and Google map methods had similar outputs with an *R*^2^ of 0.86 (peak and off peak) and the Nash-Sutcliffe model of efficiency being 0.83 (peak) and 0.76 (off-peak travel).

Model 1-a (RMH, MMC) had a greater proportion of cases arriving to hospital within 30 min in all 3 years compared with model 1-b (RMH, ALF) and 1-c (RMH, AUS) (Supplementary Table 1). In model 1-a, the median traveling time to RMH is 15 min (IQR 17.75–23.08 min), 80% of cases within idealized travel time (TT) of 30 min during inter-peak in 2016 which decline to median travel time of 20.5 min (IQR 13.8–27.3) with 72% cases within TT. The same trend can be seen in MMC from 2016 to 2026 with increase in travel time from 15 (IQR 13.3–18.13) to 18.8 (IQR 14.3–23.35) and a decrease in percentage of cases arriving under 30 min from 90 to 85%. In other 2-hub models, the general decreasing trends in coverage of nominated hospital within 30 min are observable (Supplementary Table 2). In model 1-b, the median time to RMH was 21 min (IQR 17.75–23.08) in the year 2016, 25.84 min (IQR 19.16–32.53) in the year 2021 and 26.18 min (IQR 19.43–32.92) in the year 2026; the median time to ALF was 20 min (IQR 16.54–23.15) in the year 2016, 23.98 min (IQR 16.59–31.38) in the year 2021 and 24.09 (IQR 16.65–31.53) in the year 2026. In model 1-c, the median time to RMH was 15 min (IQR13.1–18.6) in the year 2011, 19.9 min (IQR13.28–26.65) in the year 2021 and 20.5 min (IQR 13.8–27.3) in the year 2026; the median time to AUS was 15 min (IQR 13.3–18.13), 16.13 min (IQR 13.93–18.33) in the year 2021 and 18.8 min (IQR 14.3–23.35) in the year 2026.

The combination of RMH and MMC has the greatest proportion of simulated cases arriving within ideal time of 30 min, 86% (off-peak) and 82% (peak). This proportion decreases to 82% (off-peak) and 79% (peak) after 5 years and 80% (off-peak) and 77% (peak) after 10 years. The combination of RMH and ALF provides coverage for 74% of cases, 68% by 5 years and 66% by 10 year. The combination of RMH and AUS provides coverage for 70% (off-peak) and 65% (peak) of cases in the base case, 65% (off-peak) and 61% (peak) at 5 year, and 63% (off-peak) and 59% (peak) by 10 year (Table 1).

Off peak, the VITM model yields a total of 4,338 patients within MMC catchment and 5,434 patients in RMH catchment. The Google Map model yields a total of 3,854 patients within MMC and 5,958 patients. If 10% of the patients with stroke in this catchment are eligible for ECR then it is estimated from VITM model that the number of cases in the MMC and RMH catchments are 434 and 543 patients, respectively. During peak hour, the VITM model yields a total of 4,253 in MMC and 5,519 in RMH catchments. The Google Map model yields a total of 4,213 in MMC and 5,599 in RMH catchments. In this case and assuming 10% of the patients are eligible them the estimated number of cases are 425 for MMC and 552 for RMH (Supplementary Table 3).

## Discussion

The key finding from this study is that the travel time forecasts from the Google Maps API is similar to that obtained by a strategic transport model and that the two-hospital model comprising of RMH and MMC provided the optimal solution with respect to inter-peak traveling time into the future. We were able to explore future transport scenarios up to 10 years and found that this combination remains stable suggesting the RMH and MMC combination is robust in both current and future scenarios. We propose that a combination of the two methods should be used to model hospital catchment for stroke or other medical illness.

### Strategic Transport Model and the Google Maps API

The strategic transport model requires someone trained in its use and cannot be used easily by someone unfamiliar with the methodology. Running the model can take several weeks whereas the simulation with the Google Maps API can be performed overnight. Further, the license for the use of this model come from the Department of Transport and thus it is not open for public access. By contrast, the Google Maps API is open to the public upon signing up at the Google Developers' website. The two methods differ in that the main objective of strategic transport demand models is to meet long-term mobility needs on the basis of socio-economic scenario and land-use characteristics (25). As such strategic transport models like VITM produce transport metrics at the aggregate level of zone called traffic analysis zone. By contrast, the Google Maps API estimates travel time for a given trip at the specified time to individual addresses within zones. A critical difference between a strategic transport model and the Google Maps API is that the strategic transport model can be used for future travel planning. We were reassured our findings with the Google Maps API were confirmed with the strategic model using the high value on Nash-Sutcliff of model efficiency.

### Strategic Transport Model in Australia and Around the World

Similar research can be conducted for other cities. For example, in Adelaide the MASTEM (The Metropolitan Adelaide Strategic Transport Evaluation Model) (26) and the STM (The Strategic Travel Model) in Sydney can be used in a same way to define the ECR service boundaries in this City (27). In England, the London Transport Studies (LTS) (28) is available while in Zurich and Singapore, an agent based (MATsim) model is available (29).

Our study has several limitations. The focus in this paper and our earlier paper has been on travel time (18). These are other issues to consider such as the government willingness to pay and the allocated budget, the number of available accredited interventional neuroradiologists and stroke (vascular) neurologists and the observed number of stroke cases requiring ECR. For example, the requirements to apply for second designated ECR hub in Victoria included sufficient number of accredited interventional neuroradiologists (4 at MMC) and stroke neurologists (5 at MMC) and 2 angiographic suites. A coalition of 2 ECR hubs would be able to handle 4 cases simultaneously every 2 h. Such a scenario has not yet been reached. The use of VITM for predicting future scenarios are based on a number of inputs to the model and as these scenarios are estimate of future events. In this study, the term “stable” has been used to describe the lack of variation in the catchment over the years for the combination of RMH and MMC. It was 6% change in the peak traffic model for this combination and 8% decrement for the RMH and ALF and 7% decrement for RMH and AUS.

The current study does not address the issue of model of patient care such as treatment at “mothership” or treat at the local hospital first, so called “drip and ship” (8, 9). There are various arguments either way. Proponents of treatment with “direct to mothership” model would point to the better outcome with direct transfer, possibly from avoiding delay from inter-hospital transfer and earlier revascularization (10, 11). A cautious approach would be to evaluate the capacity of the “mothership” hospital to handle the diversion of all patients to the mothership before imaging. Using very conservative estimate of 10% eligible patients, the “mothership” hospital would face a deluge of patients to process to treat in order to perform ECR on 434 patients at MMC or 543 patients at RMH. A variety of tools are now available to screen patients for LVO (30, 31). However, a formal prospective field testing of these tools and the impact on hospital case load has not yet been evaluated. Prior study had suggested that evaluation of models of care should include different type of hospital ability and ambulance transport (7). We would add the use of screen tool for LVO in the modeling approach.

## Conclusion

In summary, we introduced a trip-based demand model to estimate the catchment area for ECR hubs and assess the stability of the model over time. This method can be applied in designing and planning ECR services not only in different states of Australia but also in Metropolitan cities over the world.

## Data Availability

The datasets generated for this study are available on request to the corresponding author.

## Author Contributions

TP, RB, and HV: design. AT, HV, and RB: analysis. TP, HV, RB, GC, HM, and VS: writing.

### Conflict of Interest Statement

The authors declare that the research was conducted in the absence of any commercial or financial relationships that could be construed as a potential conflict of interest.
