# Supplementary material for: Application of Strategic Transport Model and Google Maps to Develop Better Clot Retrieval Stroke Service
Source: Front Neurol. 2019 Jun 28;10:692. doi: 10.3389/fneur.2019.00692 (PMC6611389; doi:10.3389/fneur.2019.00692)
Supplement: Supplementary file 1 [file Table_1.DOCX]

Table 1: Traveling time and coverage area for different combinations of 2 ECR hub models in Melbourne, VITM vs Google Map API

| Model 1-a (RMH+MMC) Peak traffic | | | | | |
| --- | --- | --- | --- | --- | --- |
| Model | Time | RMH | | MMC | |
|  |  | Time to RMH, min | % Cases< 30 min | Time to MMC, min | % Cases< 30 min |
| VITM | 17:15 | 22.5 (IQR 17.1-28.1) | 79% | 20.7 (IQR 14.73-26.73) | 86% |
| Google Maps | 17:15 | 22 (IQR 17.3–27.4) | 82% | 19 (IQR 14.4–24.1) | 90% |
| Model 1-b (RMH+ALF) Peak traffic | | | | | |
| Model | Time | RMH | | ALF | |
|  |  | Time to RMH, min | % Cases< 30 min | Time to RMH, min | % Cases< 30 min |
| VITM | 17:15 | 25.7 (IQR 18.9-32.6) | 67% | 24 (IQR 15-33) | 65% |
| Google Maps | 17:15 | 25 (IQR 8.6–30.9) | 71% | 24 (IQR 15.9–31.8) | 69% |
| Model 1-c (RMH+AUS) Peak traffic | | | | | |
| Model | Time | RMH | | AUS | |
|  |  | Time to RMH, min | % Cases< 30 min | Time to MMC, min | % Cases< 30 min |
| VITM | 17:15 | 27 (IQR 20.3-34.6) | 70% | 20 (IQR 16.6-23.5) | 69% |
| Google Maps | 17:15 | 24 (IQR 17.9–30.8) | 72% | 24 (IQR 16.7–33.3) | 67% |
| Model 1-a (RMH+MMC) Off peak traffic | | | | | |
| Model | Time | RMH | | MMC | |
|  |  | Time to RMH, min | % Cases< 30 min | Time to MMC, min | % Cases< 30 min |
| VITM | 12:30 | 21 (IQR 15.65-27.23) | 82% | 19 (IQR 14.33-23.77) | 90% |
| Google Maps | 12:30 | 20 (IQR 16.0–26.0) | 87% | 18 (IQR 13.6–22.1) | 94% |
| Model 1-b (RMH+ALF)) Off peak traffic | | | | | |
| Model | Time | RMH | | ALF | |
|  |  | Time to RMH, min | % Cases< 30 min | Time to RMH, min | % Cases< 30 min |
| VITM | 12:30 | 21 (IQR 23.08-17.75) | 75% | 20 (IQR 16.54-23.15) | 73% |
| Google Maps | 12:30 | 23 (IQR 17.1–27.8) | 82% | 22 (IQR 17.1–27.8) | 75% |
| Model 1-c (RMH+AUS) Off peak traffic | | | | | |
| Model | Time | RMH | | AUS | |
|  |  | Time to RMH, min | % Cases< 30 min | Time to MMC, min | % Cases< 30 min |
| VITM | 12:30 | 24 (IQR 32.43-17.46) | 80% | 19 (IQR 22.68-16.31) | 75% |
| Google Maps | 12:30 | 22 (IQR 16.4–27.7) | 82% | 21 (IQR 14.9–29.5) | 76% |

RMH, Royal Melbourne Hospital; MMC, Monash Medical Centre; AUS, Austin Hospital; ALF Alfred Hospital; IQR, interquartile range

Table 2. Traveling time and proportion of patients arriving within 30 minutes based on VITM for 2 ECR hub models in future years

| Model 1-a (RMH+MMC) Peak traffic | | | | | | | | | | | |
| --- | --- | --- | --- | --- | --- | --- | --- | --- | --- | --- | --- |
| Year | Time | | RMH | | | | | MMC | | | |
|  |  |  | Coverage (km^2^) | | Time to RMH, min | % Cases< 30 min | | Coverage (km^2^) | | Time to MMC, min | % Cases< 30 min |
| 2016 | 17:15 | | 5226 | | 22.5 (IQR 17.1-28.1) | 79% | | 6010 | | 20.7 (IQR 14.73-26.73) | 86% |
| 2021 | 17:15 | | 4917 | | 23.99(IQR 18.33-29.65) | 74% | | 5826 | | 21.15(IQR 15.4-26.91) | 85% |
| 2026 | 17:15 | | 4830 | | 25.3(IQR 19.11-31.64) | 71% | | 5623 | | 21.93 (IQR 16.28-27.59 ) | 85% |
| Model 1-b (RMH+ALF) Peak traffic | | | | | | | | | | | |
| Year | Time | | RMH | | | | | ALF | | | |
|  |  |  | Coverage (km^2^) | | Time to RMH, min | % Cases< 30 min | | Coverage (km^2^) | | Time to ALF, min | % Cases< 30 min |
| 2016 | 17:15 | | 5061 | | 25.76(IQR 18.92-32.6) | 67% | | 2056 | | 24 (IQR 15-33) | 65% |
| 2021 | 17:15 | | 4628 | | 26.5(IQR 19.7-33.3) | 63% | | 1831 | | 26.2( IQR 18.3-34.1) | 63% |
| 2026 | 17:15 | | 4369 | | 27.5 (IQR 20.45-34.7) | 60% | | 1742 | | 27( IQR 19.95-34.78) | 62% |
| Model 1-c (RMH+AUS) Peak traffic | | | | | | | | | | | |
| Year | Time | | RMH | | | | | AUS | | | |
|  |  |  | Coverage (km^2^) | | Time to RMH, min | % Cases< 30 min | | Coverage (km^2^) | | Time to AUS, min | % Cases< 30 min |
| 2016 | 17:15 | | 4323 | | 27 (IQR 20.3-34.6) | 70% | | 3329 | | 20 (IQR 16.6-23.51) | 69% |
| 2021 | 17:15 | | 4069 | | 29.4(IQR 21.7-37.2) | 61% | | 2721 | | 20.8(IQR 16.9- 24.83) | 66% |
| 2026 | 17:15 | | 4121 | | 31.4 (IQR 23.6-39.3) | 53% | | 2352 | | 22.2(IQR 17.9- 26.51) | 65% |
| Model 1-a (RMH+MMC) Off peak traffic | | | | | | | | | | | |
| Model | | Time | | RMH | | | | | MMC | | |
|  |  |  |  | Time to RMH, min | | | % Cases< 30 min | | Time to MMC, min | | % Cases< 30 min |
| VITM | | 12:30 | | 21 (IQR 15.65-27.23) | | | 82% | | 19 (IQR 14.33-23.77) | | 90% |
| Google Maps | | 12:30 | | 20 (IQR 16.0–26.0) | | | 87% | | 18 (IQR 13.6–22.1) | | 94% |
| Model 1-b (RMH+ALF) Off peak traffic | | | | | | | | | | | |
| Model | | Time | | RMH | | | | | ALF | | |
|  |  |  |  | Time to RMH, min | | | % Cases< 30 min | | **Time to ALF, min** | | % Cases< 30 min |
| VITM | | 12:30 | | 21 (IQR 23.08-17.75) | | | 75% | | 20 (IQR 16.54-23.15) | | 73% |
| Google Maps | | 12:30 | | 23 (IQR 17.1–27.8) | | | 82% | | 22 (IQR 17.1–27.8) | | 75% |
| Model 1-c (RMH+AUS) Off peak traffic | | | | | | | | | | | |
| Model | | Time | | RMH | | | | | AUS | | |
|  |  |  |  | Time to RMH, min | | | % Cases< 30 min | | **Time to AUS, min** | | % Cases< 30 min |
| VITM | | 12:30 | | 24 (IQR 32.43-17.46) | | | 80% | | 19 (IQR 22.68-16.31) | | 75% |
| Google Maps | | 12:30 | | 22 (IQR 16.4–27.7) | | | 82% | | 21 (IQR 14.9–29.5) | | 76% |

RMH, Royal Melbourne Hospital; MMC, Monash Medical Centre; AUS, Austin Hospital; ALF Alfred Hospital; IQR, interquartile range

Table 3: Population in each catchment

|  | Model 1a | | Model 1b | | Model 1c | |  |
| --- | --- | --- | --- | --- | --- | --- | --- |
|  | RMH | MMC | RMH | ALF | RMH | AUS |  |
| Afternoon  Off peak | 5434 | 4338 | 5913 | 3858 | 6564 | 3202 | VITM |
|  | 5958 | 3854 | 6361 | 3453 | 4983 | 4829 | Google Map |
|  |  |  |  |  |  |  |  |
| Evening peak | 5519 | 4253 | 5696 | 4076 | 6302 | 3462 | VITM |
|  | 5599 | 4213 | 6144 | 3668 | 5315 | 4497 | Google Map |

ALF= Alfred Hospital, AUS=Austin Hospital, MMC=Monash Medical Hospital, RMH=Royal Melbourne Hospital
